# Status and Prospects of Heterojunction-Based HEMT for Next-Generation Biosensors

**DOI:** 10.3390/mi14020325

**Published:** 2023-01-27

**Authors:** Najihah Fauzi, Rahil Izzati Mohd Asri, Mohamad Faiz Mohamed Omar, Asrulnizam Abd Manaf, Hiroshi Kawarada, Shaili Falina, Mohd Syamsul

**Affiliations:** 1 Institute of Nano Optoelectronics Research and Technology (INOR), Universiti Sains Malaysia, Sains@USM, Bayan Lepas 11900, Pulau Pinang, Malaysia; 2 Collaborative Microelectronic Design Excellence Center (CEDEC), Universiti Sains Malaysia, Sains@USM, Bayan Lepas 11900, Pulau Pinang, Malaysia; 3 Faculty of Science and Engineering, Waseda University, Tokyo 169-8555, Japan; 4 The Kagami Memorial Laboratory for Materials Science and Technology, Waseda University, Nishiwaseda, Shinjuku, Tokyo 169-0051, Japan

**Keywords:** biosensor, GaN HEMT, GaAs HEMT, FET, biosensor, ISFET

## Abstract

High electron mobility transistor (HEMT) biosensors hold great potential for realizing label-free, real-time, and direct detection. Owing to their unique properties of two-dimensional electron gas (2DEG), HEMT biosensors have the ability to amplify current changes pertinent to potential changes with the introduction of any biomolecules, making them highly surface charge sensitive. This review discusses the recent advances in the use of AlGaN/GaN and AlGaAs/GaAs HEMT as biosensors in the context of different gate architectures. We describe the fundamental mechanisms underlying their operational functions, giving insight into crucial experiments as well as the necessary analysis and validation of data. Surface functionalization and biorecognition integrated into the HEMT gate structures, including self-assembly strategies, are also presented in this review, with relevant and promising applications discussed for ultra-sensitive biosensors. Obstacles and opportunities for possible optimization are also surveyed. Conclusively, future prospects for further development and applications are discussed. This review is instructive for researchers who are new to this field as well as being informative for those who work in related fields.

## 1. Introduction

In recent years, we have seen a significant outbreak of COVID-19, a disease which was both widely and rapidly spread by human-to-human transmission via droplets. Our limited technological readiness for this formidable event contributed significantly to the progression of the outbreak. Therefore, the development of a rapid and high-stability biosensor for immediate and real-time detection is essential. Biosensors have the capability of detecting ions, small chemical compounds, proteins, deoxyribonucleic acid (DNA), or ribonucleic acid (RNA), all of which exist in aqueous solutions such as water, buffered solution, blood, urine, saliva, or tears [1]. Protein-based biomarkers are extremely useful for diagnostics, particularly in cardiovascular disorders (CVDs) [2]. An effective biosensor would be able to immediately detect, identify, and quantify target biomolecules in any underlying physiological solution [3]. A biosensor device typically includes the bioreceptor, which recognizes the specific analyte and generates a signal response. While the biosensor transducer’s role is to convert biological material detected into a measurable signal, the detector also factors in signal amplification and visualization [4,5]. Generally, types of biosensors can be classified based on their signal transducer systems, such as electrochemical, optical, thermal, and piezoelectric biosensors [6]. 

Field-effect transistor (FET)-based sensors have been broadly researched for the detection of biological molecules owing to their distinct advantages and exclusive potential features. FET-based biosensors offer many advantages, including fast response time, label-free detection, and excellent sensitivity [7,8,9,10]. Moreover, their ability to be extremely sensitive, provide instantaneous measurements, and allow for the efficient detection of analytes in low concentrations makes them a favourable device for biosensors [11]. The biological interaction takes place at the sensing gate region, which modifies the surface potential differential and the current channel between the source and drain [7]. The biological interactions are then amplified into electrical signals [12]. With further modification of the sensing gate region, FET biosensors can be highly specific and selective for their respective target analytes [13]. There are many types of FET biosensors utilizing advanced materials, including transition metal dichalcogenides (TMDCs)-based FETs [14,15], graphene FETs [16,17], diamond FETs [18,19], and others. Concurrently, III–V semiconductor-based FETs, which are also customarily known as high-electron-mobility transistor (HEMT) devices, have been extensively used in a variety of applications such as power devices [20,21], sensors [22,23], circuits [24], or as a complementary device for light-emitting diodes (LEDs) [25] owing to their high mobility and high concentration of 2-dimensional electron gas (2-DEG) [26] properties. Gallium nitride (GaN) and gallium arsenide (GaAs) are both III-V compounds that are commonly used as HEMT materials. HEMT-based biosensors have attracted significant attention from researchers in this field because they have high surface charge sensitivity [27,28]. Various design improvements have been developed in recent years to overcome the limitations of these heterojunction-based HEMTs as biosensors in order to achieve their optimum potential. These structural design improvements and advancements were thoroughly investigated to determine which could provide better stability and sensitivity to the biosensor [29,30,31].

HEMT sensors have been extensively studied for over a decade, and many review articles have been published on HEMT biosensors, signifying their progressive development in this field. In 2015, Kirste et al. wrote a thorough review of the potential of III-nitride semiconductor materials for electronic biosensors [32]. Sarangadharan et al. discussed in detail the topic of HEMT biosensor development, specifically for detection in physiological environments such as 1 X PBS/whole blood/serum/cell tissues [33]. Gudkov et al. provide brilliant insights pertaining to previous HEMT biosensor technology and perspectives on future generations of low-cost biosensors in their short review article [4]. Recently, Hemaja et al. published a comprehensive review on recent advances of HEMT-based biosensors, in which they included a comparative analysis of GaN-based HEMT with silicon-based sensors [34]. Despite the extensive progress that has been charted in HEMT devices for biosensors; however, hardly any reviews have particularly discussed the properties of each type of gate structure of HEMT biosensors in depth yet. Besides channel engineering, the selection of appropriate sensing elements, and intrinsic parameter optimization, the next important aspect that needs to be considered in HEMT biosensor fabrication devices is the gate structure. Gate structure determines the complexity of the sensor device fabrication process, which influences the sensor device cost and some of the sensor’s performance matrices. 

This review aims to provide an overview of heterojunction-based HEMT biosensors with the different gate structures that have been reported in the literature over the past 10 years (from 2012 to present). This review begins by describing the HEMT biosensor’s configuration and characteristics. The following sections discuss the principles, unique properties, and sensing mechanisms of each type of HEMT biosensor gate structure. The gate structures of HEMT biosensors presented in this review include electrolyte gate, followed by extended gate, electric double-layer (EDL) gate, gateless, floating-gate, and dual-gate structures. Furthermore, this review also outlines the HEMT biosensor challenges or difficulties in real-life implementation. The authors would like to apologize in advance for any potential exclusion of additional key works in this field.

## 2. Heterojunction-Based HEMT as Biosensor

**Figure 1:**
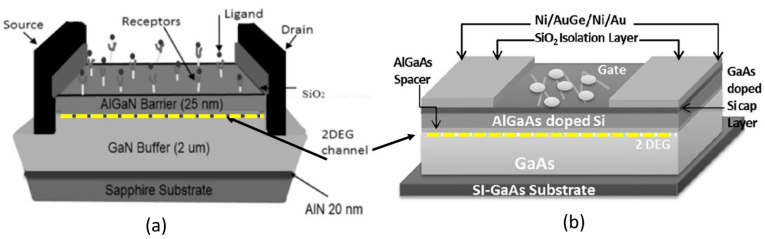
Schematic diagram of (**a**) AlGaN/GaN HEMT and (**b**) AlGaAs/GaAs HEMT with 2DEG (marked with dashed lines). Copyright 2021, with permission from Elsevier [38] and copyright 2021, with permission from Elsevier [49], respectively.

Quantitative electrical detection provided by HEMT-based sensors appears to be the most promising solution for real-time and highly sensitive sensors. On that account, HEMT has been extensively studied to detect a variety of ions and biomolecules [35,36,37,38,39,40,41]. The construction of HEMT biosensors is analogous to that of FET biosensors, where it basically has three terminals; source, drain, and gate. The source and drain terminals are bridged by high-density two-dimensional electron gas (2DEG) underneath the HEMT sensing area (channel). The 2DEG is responsible for the conductivity of the HEMT sensor and is characterized by a high electron sheet carrier concentration, where it can be modulated by the gate voltage [42]. In the case of AlGaN/GaN HEMT, the 2DEG is induced by piezoelectric polarization of the strained AlGaN layer together with spontaneous polarization of GaN and the AlGaN layer [43,44,45]. In addition to its remarkable 2DEG with high carrier densities (~10^6^ to 10^13^ cm^−2^), HEMT sensors are also reported to have high saturation velocities of from 1.2 to 1.5 × 10^7^ cm/s, and high electron mobilities from 1500 cm^2^ V^−1^s^−1^ to 8500 cm^2^ V^−1^s^−1^ [38,46,47,48]. Figure 1a,b show AlGaN/GaN and AlGaAs/GaAs HEMT sensor device configurations, respectively. 

As aforementioned, the construction of an HEMT biosensor is similar to that of a FET biosensor. Although the HEMT biosensor has demonstrated better sensing performance compared to the FET biosensor, this is owing to its sensing surface, which is extremely sensitive to any minor charge changes due to the location of the 2DEG channel that is near the sensing surface [50]. Essentially, the HEMT device is already sensitive even without any functionalization on the sensing surface [51,52], whereas the majority of FET biosensors require surface functionalization for sensing purposes [53]. Additionally, HEMT is a normally on operation device [38] and does not require a gate electrode to turn on, which contrasts with the conventional FET [39,40]. Therefore, the fact that the reference electrode is not a necessity becomes a significant benefit of using HEMTs, which are easier for device miniaturization and integration [41]. 

So far, however, the HEMT sensor surface area has not been closely examined. Nevertheless, with appropriate functionalization on the channel surface, the surface area of the HEMT biosensor can be enhanced [54]. A key advantage of the HEMT biosensor over other biosensors is that it allows for rapid direct detection in physiological salt environments without the need for sample pre-treatments. Generally, the conventional FET biosensors require sample dilution or filtration before detection. As shown in Table 1, HEMT-based biosensors demonstrated a remarkable rapid detection time in combination with a highly sensitive sensing performance. In addition, data from several studies in Table 2 suggest that the HEMT biosensor exhibits good reproducibility rates with relative standard deviations (RSDs) below 10%. 

## 3. Electrolyte Gate HEMT

**Figure 2:**
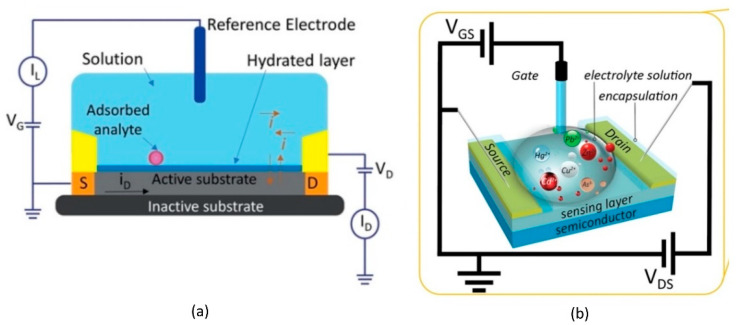
Illustration of (**a**) basic structure of electrolyte-gate and (**b**) three-dimensional image of HEMT biosensor. Reprinted from reference [93]. Copyright 2021, with permission from MDPI.

Fundamentally, all of the biomolecules require aqueous media for their proper functioning. Electrolyte-gated HEMTs have become prominent as building blocks for biomolecules because they are stable in aqueous environments and are able to transduce and amplify the biological signal into an electrical signal at a low voltage [90,91]. In the basic principles of electrolyte-gate HEMT, the channel and the external reference electrode are in direct contact with a target electrolyte. An Ag/AgCl reference electrode or other external reference electrode (e.g., a platinum (Pt) reference electrode) is commonly used to modulate the sensor device channel conductance [92]. The polarity and magnitude of the gate voltage will drift the anions or cations in the electrolyte to the channel surface, resulting in the depletion or enhancement of the 2DEG numbers underneath the channel. The changes in 2DEG in the channel are reflected in channel conductivity variation, which, in turn, modulates the drain current (I_D_) flowing through the HEMT channel. This particular section is dedicated for HEMT biosensors with electrolyte-gate basic structures as illustrated in Figure 2a,b.

To augment the sensitivity of the HEMT biosensor, modification of the HEMT surface is necessary. Maeda et al. investigated the use of an AlGaN cap layer and the incorporation of aluminium (Al) composition in the AlGaN layer in relation to pH sensitivity [94]. In order to understand how these two parameters affected pH sensitivity, the authors prepared seven AlGaN/GaN heterostructures, of which four of them were incorporated with different Al compositions of 22%, 24%, 25%, and 35% in the AlGaN layer, but none of them was employed with the cap layer. The other three AlGaN/GaN heterostructure samples were incorporated with 24% of the Al composition in the AlGaN layer, and the cap layer of i-GaN, p-GaN, and n-GaN was employed on each of the samples. The results show the average sensitivity of the samples with i-GaN, p-GaN, and n-GaN cap layers on the HEMT surface are 49.5, 47.2, and 51.8 mV/pH, respectively. Meanwhile, the sensitivity of the pH sensor shows an increasing trend with the increasing Al composition. Thus, the AlGaN surfaces with the highest Al composition of 35% exhibited the highest sensitivity of 55.2 mV/pH. This finding seems reasonable, as increasing the Al composition is basically increasing the functionalization molecules on the AlGaN surface, consequently increasing the sensitivity of the surface towards hydrogen ions. This finding broadly supports the work of other studies that link the use of metal oxides on ion-sensitive field-effect transistors (ISFETs) for pH sensitivity enhancements [95,96,97]. Somehow, this study is limited by the lack of information on the impact of the i-GaN, p-GaN, and n-GaN cap layers corresponding to the pH sensitivity of the sensor. In recent work, Sharma et al. fabricated n-type-doped AlGaN/GaN HEMTs-based sensor for pH and salinity sensing [98]. This study contributes to the limited research on AlGaN/GaN HEMT sensor response at varying pH levels and diverse molar concentrations of salty liquids. The pH response of the proposed sensor was evaluated in phosphate-buffered saline (PBS) solution at different pH values (pH 4, pH 7, and pH 10). The experimental pH sensing results show the output current drain decreased linearly with the increasing pH values, and the sensitivity of the sensor device in the acidic region was higher than in the basic region. The pH sensitivities in the acidic region (pH 4–pH 7) and basic region (pH 7–pH 10) were 7.783 μA/mm-pH and 0.66 μA/mm-pH, respectively. The authors hypothesized that the lower pH response observed in the basic region was caused by a higher magnitude of pH deteriorating the sensor surface. The same HEMT sensor yielded an exceptional pH sensitivity of 6.48 mA/mm-molar of NaCl in deionized (DI) water recorded at V_ds_ = 1 V and V_gs_ = 0 V, whereas the pH sensitivity of NaCl in PBS solution was 2.02 mA/mm-molar recorded at V_ds_ = 5 V and V_gs_ = 0 V. These results demonstrated the sensor’s remarkable response to relatively small variations of salt concentration in a solution. Furthermore, the device’s good response time to changes in molar concentration was retrieved as being between 250 and 350 ms. Taken together, the aforementioned findings show that AlGaN/GaN HEMT devices are extremely promising, offering high-level sensitivity pH and salinity sensors for chemical detection in biomedical applications. Wang et al. examined the effect of oxygen (O_2_) plasma treatment with different times of exposure (2 min vs. 30 min) on the performance of AlGaN/GaN HEMT sensors [99]. The results show that the sensitivity of the AlGaN/GaN ISFET improved to 55.7 mV/pH for short-time O_2_ plasma treatment. The AlGaN/GaN HEMT surface was observed to be significantly hydrophilic, with a water contact angle of around 5–7°, and it is also much smoother and cleaner. According to the their X-ray photoelectron spectroscopy (XPS) report, interestingly, the AlGaN/GaN surface was rich with aluminum oxide (Al₂O₃). Evidently, the Al₂O₃ was formed during the short exposure to O_2_ plasma. Because of this reason, the pH sensitivity of AlGaN/GaN HEMT was enhanced significantly. In contrast, as exposure time increased to 30 min, the Al₂O₃ dominant AlGaN/GaN surface shifted to a gallium oxide-dominated surface, resulting in a decrease in ISFET sensitivity to 41.1 mV/pH. This finding, while preliminary, suggests that a short duration O_2_ treatment is an alternative and simple strategy to improve the pH sensitivity of AlGaN/GaN HEMT. An additional study will be needed to optimize the most effective exposure time for O_2_ plasma on AlGaN/GaN HEMT to yield maximum pH sensitivity. Exploiting the same idea, Xue et al. explored the surface hydroxylation treatment on AlGaN/GaN HEMT-based surfaces for H^+^ detection [100]. The experiment was carried out by dipping the GaN samples at 80 °C for 20 min in a mixture of 98% concentrated sulfuric acid and 30% hydrogen peroxide solution (volume mixing was 3:1). Note that this treatment was abbreviated as SPM. The water contact angle on GaN was found to be reduced from 41 to 9°, the RMS roughness of the GaN surface decreased from 0.75 nm to 0.70 nm, and the density of hydroxyl groups on the GaN surface increased to approximately 5 times that of the original condition. Moreover, the sensitivity of the treated GaN sample increased to 113.3 μA/pH from 46.7 μA/pH. The sensor device demonstrated good repeatability before and after SPM treatment at different pH values. In accordance with these results, previous studies have demonstrated that depositing oxide layers or surface oxidation would increase the surface hydroxyl binding site, consequently improving the device’s response towards H^+^ [94,101]. SPM treatment might effectively increase the number of hydroxyl groups on the surface, dramatically improving hydrophilicity and surface morphology. This study has proven that the SPM treatment could efficiently enhance the repeatability, stability, and sensitivity of the biosensor owing to the increasing number of surface H^+^ binding sites. 

**Figure 3:**
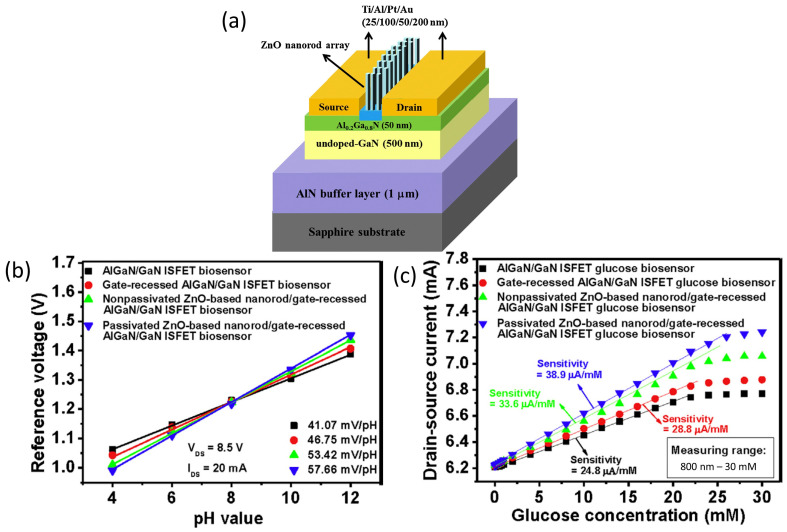
(**a**) Illustration of cross-sectional area of ZnO nanorod array-based HEMT sensor. (**b**) Reference voltage as a function of pH values and (**c**) drain-source current as a function of glucose concentration. Reprinted from reference [102]. Copyright 2015, with permission from Elsevier.

Lee et al. reported an innovative technique for AlGaN/GaN HEMT gate fabrication and surface functionalization, using photoelectrochemical (PEC) methods to achieve a highly sensitive glucose sensor [102]. Lee’s work was primarily focused on the construction of a recess gate to improve the AlGaN/GaN HEMT sensor performance. The PEC etching method was used to etch the AlGaN layer to form the recessed gate structure, followed by the direct growth of an insulator on the gate region using the PEC oxidation method. Conversely, for the glucose sensing membrane, a Zinc oxide (ZnO) nanorod array was grown on the AlGaN/GaN heterostructure, and subsequently, the PEC passivation method was applied onto the nanorod array to reduce the dangling bonds. The structure of the sensor device was shown in Figure 3a. Overall, this sensor fabrication approach has improved the sensing performance of the AlGaN/GaN HEMT sensor. The resulting AlGaN/GAN HEMT sensor demonstrated pH sensitivity in a nearly Nernstian response of 57.66 mV/pH and a wide range glucose detection in a concentration range from 800 nM to 25 mM, with a sensitivity of 38.9 μA/mM, as presented in the graph in Figure 3b,c, respectively. This HEMT sensing enhancement was attributed to the gate-recessed structure etched by the PEC etching process, which shortened the distance between the sensing surface and the 2DEG channel layer and, at the same time, improved the channel controlling ability of the gate region. Furthermore, the gate insulator generated via PEC oxidation could increase the quality of the interface between the gate insulator and the semiconductor, while the PEC passivation approach used on ZnO nanorods could minimize dangling bonds and surface states on the sidewall surface, which can preserve the affinity of the enzyme. This study provided a deeper insight into the PEC method in order to improve the performance of the AlGaN/GaN ISFET and the ZnO nanorod sensing membrane. 

One of the most highly desirable properties of a biosensor is that it should be a simple and sensitive sensor system that has a compact dimension for portability. A high-performance AlGaN/GaN HEMT device combined with a null-balancing circuit has been proposed to detect C-reactive protein (CRP) [103]. The integration of the null-balancing circuit into this sensor system has simplified the biosensor system measurement without the need for a bulky semiconductor parametric analyzer (SPA). Moreover, the integrated system also offers more stable output with noise cancellation [104]. This HEMT biosensor sensing area was fabricated with SAM on the Ni/Au gate region. The negatively charged thiolated surface Au gate region enabled binding with the positively charge anti-CRP receptor. As the CRP target was introduced to the sensing area, changes were caused in the net charges of the gate region. This change is directly measured by the sensor’s output voltage instead of the drain current of the device. This sensor exhibits great selectivity over a broad sensing range from 10 ng/mL to 1000 ng/mL. Other examples of electrolyte-gate HEMT biosensors are summarized in Table 3. 

## 4. Extended Gate HEMT

**Figure 4:**
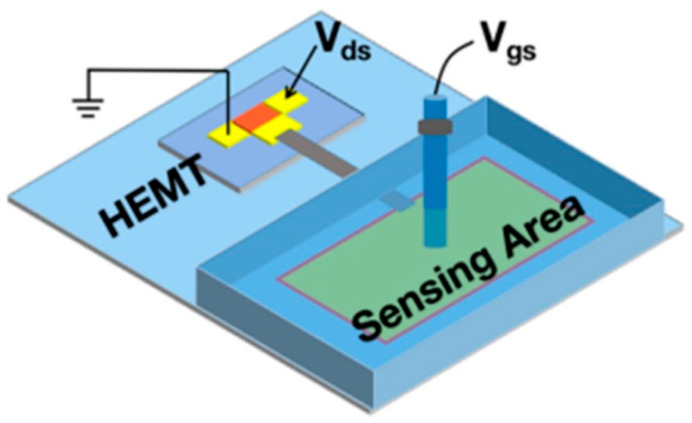
The example structure of an extended gate biosensor. Reprinted from reference [125]. Copyright 2022, with permission from Elsevier.

One major issue that has dominated the field of sensors for many years concerns the reproducibility and reliability of sensor devices. An HEMT biosensor is a kind of transistor that works in electrolytes. To be able to conduct measurements in a solution, the sensor device requires excellent passivation to avoid the electrolyte from penetrating into the device’s circuitry. Similar to the FET sensor, the sensing area of the conventional structure of an AlGaN/GaN HEMT sensor is positioned between the source and drain electrodes, resulting in poor isolation between the device and biological environment. To solve the isolation problem, several fabrication methods have been demonstrated. Sarangadharan et al. passivated the entire HEMT sensor device using photoresist, and later the openings were made at the sensing region and the gate metal using photolithography [120]. An ideal passivation layer should be as thin as possible in order to perpetuate the sensor’s sensitivity at the sensing area. On that account, many researchers in this field have explored the deposition of self-assembled monolayers (SAM) of alkanethiols [121,122,123]. However, one prominent disadvantage of this passivation method is that it is often incomplete [123]. An innovative passivation strategy involving the formation of a monolayer of (3-mercaptopropyl)-trimethoxysilane (MPT) and the subsequent polymerization of the trimethoxysilyl-terminated surface has been introduced by Sileo et al. group [124]. However, the fabrication process of these passivation techniques is still too complex. An extended gate HEMT structure is one of the options where the complex passivation process can be eliminated in the HEMT biosensor fabrication. The extended gate HEMT structure became a promising solution for many researchers to overcome the drawback of poor isolation on HEMT biosensors by proposing a separate transducer (HEMT devices) and sensing membrane (extended gate). This configuration completely isolates the chemical and biological environment from the HEMT circuitry. Thus, during sensor assessment, only the extended electrodes with the sensing gate come into contact with the solution, assuring the long-term sensitivity and stability of sensors. Although the sensing membrane has been contaminated or destroyed, the transducer can be reused. This structure is also called a disposable sensor, as the sensing membrane can be easily implemented and repackaged. Therefore, this design provides simple packaging that has flexibility for testing and characterization without contact with the solution. Figure 4 below shows an example structure of an extended gate biosensor. In this section, various extended gate structures are reviewed for different sensor applications. 

**Figure 5:**
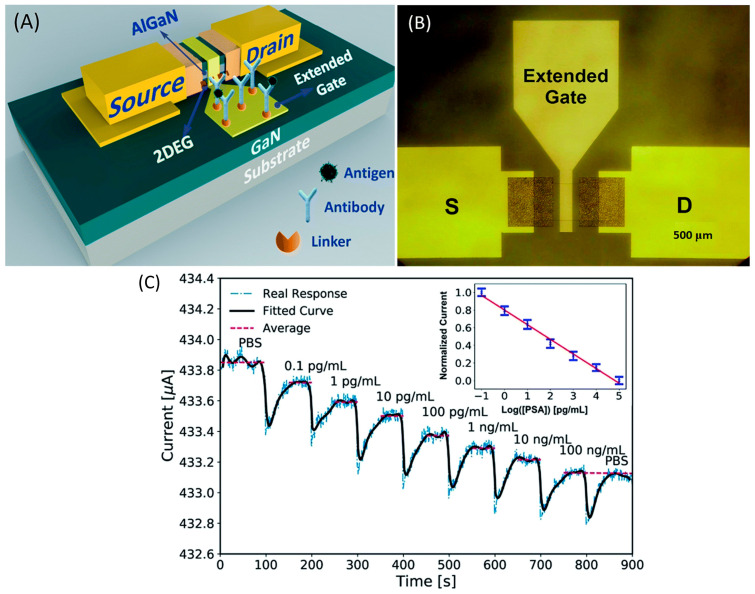
The structure of the EG-AlGaN/GaN HEMT: (**A**) a schematic illustration of the device, and (**B**) a plan view photomicrograph. (**C**) The real-time detection of PSA (the bold black line is a fitted curve and the red dashed lines are the average values). Reprinted with permission from [126]. Copyright 2017, RSC Advances.

**Figure 6:**
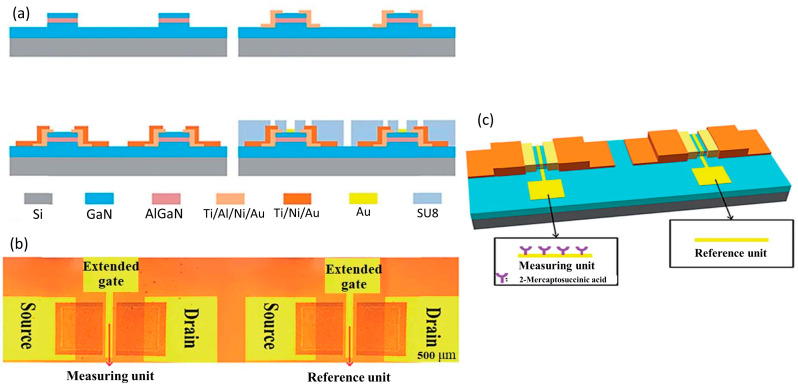
Illustration of (**a**) the fabrication process (**b**) the real image and (**c**) the three dimensional structure of the differential HEMT sensor. Reprinted from reference [128]. Copyright 2019, with permission from The Royal Society of Chemistry.

Ding’s group investigated an extended gate-AlGaN/GaN HEMT for detecting a prostate-specific antigen (PSA) as a biomarker for prostate cancer [126]. In this work, the research group has adopted the gold extended gate as a sensing region with a larger size of 700 µm × 700 µm to amplify the sensing signal as shown in Figure 5a,b. The sensing region was modified to form ubiquitous thiol–gold bonds before being immobilized with monoclonal antibodies as receptors to capture the PSA by immersion in deoxygenated cysteamine solution for 6 *h* at room temperature. It has been reported that modification of the gold layer prepared by this method may degrade the performance of AlGaN/GaN HEMT transducers [127]; however, contrary to expectations, this study found that the gold layer on the sensing region had less influence on the HEMT sensing performance. Figure 6c illustrated that the current response was very consistent for the first 100 s after the addition of PBS, which demonstrated the stability of the device. Besides, this device also displays its specificity when a current response quickly returns to its previous level (i.e., at a PSA concentration of 100 ng/mL) after a drop of PBS was injected at the end of the detection. Their results showed significant current response of the transducer with the larger sensing region. A possible explanation for this might be that the larger sensing region can accommodate more surface receptors, thereby leading to enhanced sensing performance. The sensor exhibited high sensitivity and selectivity of PSA with a limit detection of 0.1 pg/mL and a wide range detection of 0.1 pg/mL to 100 ng/mL, as shown in Figure 5c.

The conventional AlGaN/GaN HEMT sensor is compromised by noise factors in experiments, thereby leading to imprecision in the output signal. Moreover, the smaller sensing area on the conventional HEMT biosensor structure results in lower sensing performances. To overcome this problem, Zhao et al. brilliantly developed a novel differential extended gate AlGaN/GaN HEMT for the real-time detection of ionic pollutant Fe^3+^ [128]. The HEMT sensor was fabricated with two extended gate sensing units: one for the measuring unit and the other for the reference unit, as shown in Figure 6a,b. On the measuring gate unit, they functionalized 2-mercaptosuccinic acid to be selectively complexed with Fe^3+^. Meanwhile, the reference gate unit was operating in a differential mode to reduce common signal (noise). The illustration of this HEMT sensor configuration is shown in Figure 6c. This HEMT biosensor configuration has several advantages, such as providing good isolation between the device and the biological environment, having a larger sensing area, and combining differential mode technology to reduce noise factors in the measurement. Their findings show that there are abrupt changes in source–drain current when Fe^3+^ is introduced into the target solution. This result implies that the sensor has exceptional specificity for Fe^3+^ detection. In addition, the proposed sensor exhibited an excellent detection limit of 10 fM, which is superior to the results of a previously reported Fe^3+^ sensor that demonstrated a LOD between 50 fM–5 μM [129,130,131,132]. The dynamic sensing range was 10 fM to 100 μM, with R^2^ = 0.9955 for linearity. The result of this study showed that this unique and novel HEMT configuration can significantly improve the sensing performance of the sensor and overcome the drawbacks of conventional HEMT biosensors. 

Extended-gate HEMT has also been proposed as a pH detection sensor. Pyo et al. demonstrated a pH EG-HEMT sensor by connecting the sensing structure, fabricated with tin dioxide (SnO_2_), to an HEMT device [133]. It is noteworthy to mention that the HEMT was constructed using metal-insulator semiconductor (MIS) structure to overcome the unstable gate leakage of conventional metal-semiconductor (MS) HEMT structures. As a result, the sensor exhibited stable HEMT operation with high output characteristics of current–voltage (I_D_-V_D_) curves. Furthermore, the resulting sensor has shown a linear pH response (pH 3 to pH 10), with an excellent sensitivity of 57.6 mV/pH, which is close to the Nernstian limit (59 mV/pH). The sensitivity of this SnO_2_ EG-HEMT pH sensor was comparable to that of an SnO_2_ film-based pH EGFET sensor by Chi et al., where its pH sensitivity was 56–58 mV/pH in the range of pH 2 to pH 12 [134]. Their experiment data conclusively show that the proposed sensor has a small measurement error of 2.39%, indicating the sensor has demonstrated outstanding stability and reliability.

Similarly, Chou et al. also found that SnO_2_-based EGFET was fabricated using sol–gel technology, resulting in a comparable pH sensitivity of 57.63 mV/pH in the pH range of pH 1 to pH 9 [135]. Although HEMT is well known as a superior sensor device compared to FET owing to its dense electron accumulation near the surface, in these cases, however, the detection of hydroxyl (OH^−^) and hydrogen (H^+^) ions in pH solutions takes place on the extended gate, which is separate from the HEMT or FET sensor surface. Thereby, the pH sensitivity is possibly determined by the SnO_2_ sensing membrane. That being said, the extended gate HEMT may benefit from high electron mobility and high current on/off ratio, which play important roles in sensor response time. The SnO_2_ EG-HEMT by Pyo et al. demonstrated an acceptable response time for pH detection (60 s/pH). In a different study, ethanolamine (EA)-modified ZnO nanorod (ZnO NRs)-based extended AlGaN/GaN HEMT was proposed for pH sensing [86]. The EA modification of ZnO NRs provided a dense monolayer amine (-NH_2_) coating of the sensing region, which was useful to protect the ZnO NRs from being corroded in strong acidic and alkaline solutions, consequently allowing for detection in a wider pH range. On top of that, the resulting sensor also showed an enhancement in pH sensitivity compared to the sensor with bare ZnO NRs. A possible explanation that promotes the pH sensor’s sensitivity is because of the versatility and ability of NH_2_ to protonate and deprotonate at different pH solutions, leading to ZnO NRs surface potential change. In this case, the change potential at the ZnO NRs extended gate will be transferred to the gate region of the AlGan/GaN HEMT. The sensitivities of the pH sensor were 22.231 µA/pH and 19.561 µA/pH at pH = 1.76–4.12 (acidic) and pH = 9.16–10.18, respectively.

In a study conducted by Xu et al. [136], an extended gate AlGaAs/GaAs HEMT sensor was fabricated to create a highly sensitive and reusable label-free biosensor to reliably detect cardiac troponin I antigen (cTnI) at clinically important ranges in both whole and diluted human serum. The gold (Au) extended gate (sensing area) was modified with cTnI antibodies to selectively bind with the cTnI antigen. In an attempt to enhance the sensitivity of their sensor, Xu and colleagues used bovine serum albumin (BSA) blocking reagent after the immobilization of the cTnI on the Au gate to reduce the non-specific binding with the sensing area. The implementation of BSA in the experiment evidently improved sensor performances. The results provided a much wider linear range of detection (100 fg/mL to 1 ng/mL) compared to the detection of cnTI without BSA blocking, which had values of 0.5 pg/mL to 50 pg/mL. The limit of detection was achieved as low as 100 fg/mL. Additionally, the authors investigated the effect of blocking molecules’ sizes in relation to the sensor’s accuracy. Their findings highlighted that the blocking reagents, whose molecules’ sizes are as large as the target molecules, have a smaller relative error (about 4%), and thereby the sensor’s accuracy can be significantly improved by choosing the appropriate blocking molecules. In a real sample analysis of cTnI in human serum, the HEMT bioassay demonstrated linear detection in the range from 15 pg/mL to 200 pg/mL with a relative error smaller than 10%. Further, the following year, Xu and colleagues carried out a series of experiments that continued from the previous studies. This study was focused on the effect of sensing area in relation to the upper LOD of cTnI antigen using HEMT bioassay [28]. The Au sensing pad (extended gate) was enlarged to 50 times the size of the gate HEMT. Xu and colleagues reported a linear relationship between the upper LOD and the size of the sensing area. A larger size of the sensing pad could provide more space for antibody–antigen binding, yielding a larger electrical signal and, at the same time, broadening the detection range. With 50 times the size gate HEMT, the upper LOD was expanded to 10 μg/mL, which is much broader than the commercial cTnI sensor that has been used in clinical detection in hospitals (Unicel Dx1800). Another example of extended-gate AlGaAs/GaAs HEMT biosensor was presented by Yu et al. [137]. In this study, a facile, highly selective, low-cost, and label-free HEMT bioassay was employed with an extended electric double-layer (EDL) gate to detect the presence of prostate-specific antigen (PSA). The novel extended gate was fabricated in a single pair separated by 1 mm space interval. The fabrication of the extended gate commences with the evaporation of a 40 nm titanium (Ti) layer on a cheap glass (substrate), followed by a 60 nm layer of Au on top of the Ti layer. One of the extended gates (with an area of 5 *×* 7 mm^2^) was functionalized with anti-PSA antibody to function as a sensing pad. Whereas the other extended gate (dimension unknown) was used as a metal pad to apply gate bias (V_GS_), the dropwise application of PSA antigens on these extended gates would bridge the gold electrode pair. However, caution must be exercised when dropping the PSA antigen sample on the extended gates to avoid the sample solution contacting the gate bias probe. The underlying sensing mechanism of this bioassay is based on capacitance change upon biorecognition on the extended sensing gate. When the gate bias is applied on the Au pad, the free mobile ions will migrate under the electric field to form an EDL at each of the electrodes. These two parallel layers of charge are virtually seen as a capacitor (thus, it is also known as EDL capacitance, C_s_) positioned in series with the HEMT device capacitance (C_d_). In this respect, the binding of the antibody and the antigen will induce the C_s_, consequently modulating the drain current (I_D_) of the HEMT sensor. The results demonstrated the threshold voltage, V_TH_ of the HEMT sensor device, shifting linearly with the increase in PSA concentration, suggesting current changes upon the detection of PSA. This circumstance was observed in a wide range of outcomes, from 100 fg/mL to 10 ng/mL in both 0.1 X and 1 X phosphate-buffered saline (PBS) solutions. The maximum sensitivity of the HEMT bioassay for the detection of 10 ng/mL PSA in 0.1 X and 1 X PBS was 55% and 35%, respectively. On a related note, the detection of PSA in 1 X PBS was performed to mimic the physiological environments of human serum. In the specificity test analysis, the proposed sensor exhibited good specificity, despite the presence of interference biomolecules (MiR-208a). This study raised the possibility that the proposed HEMT bioassay with the separative extended-EDL gate can be used for direct detection of PSA, although future work is required to optimize the HEMT sensor’s sensitivity to at least 50% in 1 X PBS solution. 

## 5. Electric Double-Layers (EDL HEMT)

**Figure 7:**
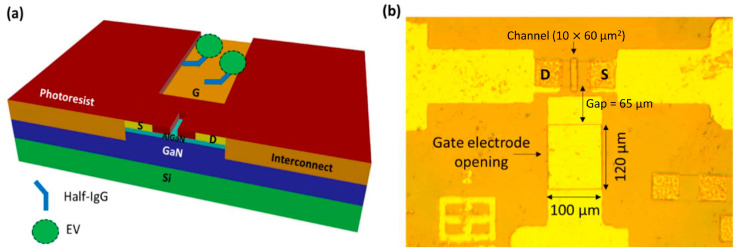
The schematics (**a**) side and (**b**) top view of EDL HEMT. Reprinted from reference [139]. Copyright 2018, with permission from *Sensors* and *Materials*.

Electric double-layer (EDL)-gated HEMT have been extensively explored in biosensing platforms since they have the benefit of the direct detection of samples in physiological concentrations [120,138]. The EDL structure has the active channel between the source and drain metals and the gate electrode all on the same plane, but the sensing area in the gate electrode is spatially separated from the active channel of HEMTs. The schematic side and top views of EDL HEMT are shown in Figure 7a,b, with the gate electrode and HEMT channel being selectively exposed using photolithography [139].

The structure is described as follows: the sample solution deposited on the sensor simulates a liquid capacitor, with two conducting plates (the HEMT channel and gate electrode) sandwiched by a dielectric medium (test solution). Here, the sample solution is employed to act as an additional dielectric in the sensor system [140]. When the gate voltage is applied, mobile ions in the sample solution instantly polarize, resulting in the formation of EDL on both the gate surface and the active channel interface of HEMT. The charge distribution at EDL generates a solution capacitance, C_s_. Thus, any changes in the C_s_ will regulate a potential drop in the device dielectric medium, hence resulting in the current output response. This is due to the charge redistribution changes at the EDL [141]. In fact, the C_s_ can change in several states; (1) when the ionic strength of the test medium changes, (2) when the surface area of the gate electrode is functionalized, and (3) when receptor–ligand binding modifies the electrostatic interaction at the gate electrode’s EDL [142]. The behaviour of EDL HEMTs can be explained by the current gain from the overall capacitance changes that relies on the difference in drain current before and after applying the gate bias. The use of current gain as a sensor index offers better stability than using absolute drain current, which may fluctuate throughout the measurements [143]. The sensing mechanism occurs when the positive bias is applied at the gate electrode, and the negative ions accumulate at the surface of the gate electrode. Simultaneously, positive ions will gather on the active channel’s surface, which leads to an increase in 2DEG concentration in the active channel of HEMT to balance out the charges and results in a sensor’s gain [144]. 

**Figure 8:**
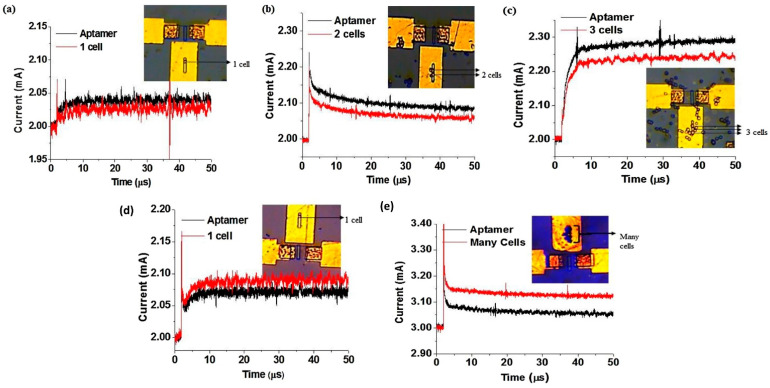
Change in drain current gain upon aptamer immobilization tested in 1 X PBS (**a**) 1 cell (**b**) 2 cells (**c**) 3 cells. Change in drain current in cell culture medium (**d**) 1 cell and (**e**) many cells captured by the aptamer. Reproduced with permission from reference [145]. Copyright 2018, Elsevier.

Kumar’s group demonstrated an EDL-gated AlGaN/GaN HEMT-based biosensor array for circulating tumor cells (CTCs) detection in a small sample volume [145]. In this study, a CTC-specific aptamer is used as a receptor and immobilized on the gold gate electrode area. The aptamer is thiolate at one end to enable covalent linking and the formation of self-assembled monolayer (SAM) on the gold substrate via the stable gold-thiol surface chemistry. The current gain decreases upon successful binding with aptamer in 1 X PBS. When the transistor is biased, the surface modification of the gate electrode alters the local charge distribution within the EDL on the gate electrode and the channel, which in turn affects the solution capacitance. As can be observed in Figure 8a,b,d, the current gain with respect to the aptamer baseline decreases further as a greater number of cells are captured on the gate electrode. What makes this finding interesting for single-cell detection is that the the current gain drops by about 2.02 mA from its initial value of 2.04 mA (aptamer). In the scale of 0.1 mA, we can deduce that a 20% decrease occurs with single-cell binding. Further, with the detection of 2 cells, the current gain drops by 20% on the same scale of 0.1 mA (2.09 mA to 2.06 mA), and with 3 cells, the current gain drops from 2.29 mA to 2.25 mA. Conversely, in cell culture medium, Figure 8d,e shows the change in current gain increases in the cell culture medium as more cells are captured. The sensitivity and absolute value of change in current gain are very similar for both environments, hence the only difference is the direction of change in current gain (towards negative or positive current). These results signify that the EDL device is able to detect up to single-cell resolution by magnifying the current gain and signal-to-noise ratio from a specific transduction event. Additionally, this sensor also affirms specificity when there is no significant change in current gain without aptamer immobilization, indicating that other residues or cells present in the background medium do not affect the sensor signal. Further, they develop a sensor array and microfluidic channel with a simple polymer. This is suitable for miniaturization, with the HEMT sensors maximizing output with simultaneous detections at multiple sites on the same chip. These findings verify that EDL gate HEMT can provide good selectivity, specificity, and sensitivity as a sensor without the need for any sample pre-processing such as electrolyte dilution, filtering, or desalting. Despite its remarkable accomplishment, further research is required to examine the range of detection and LOD for this sensor. 

Later, they the researchers investigated the transmembrane potential changes of CTC cells with the same EDL sensor [146]. The transmembrane potential response was studied by modulating the concentration of divalent cations such as Ca^2+^ and Mg^2+^ since they provide stimuli to CTCs, leading to a cellular response that results in sensor signal changes. The absence of either of the divalent cations alters the transmembrane potential from its equilibrium state towards a more depolarized potential. Following the result, when CTCs are suspended in HBSS without Ca^2+^ or Mg^2+^, the local charge distribution in the EDL changes, altering the C_s_ of the sensor and resulting in current decreases. This is due to the imbalance in the electrochemical and ionic gradient triggered by the extracellular and intracellular distribution of ions, resulting in a shift in transmembrane potential. These findings validate that the EDL HEMT platform can be used to monitor membrane potential changes in a single cell without requiring extensive calibration procedures.

**Figure 9:**
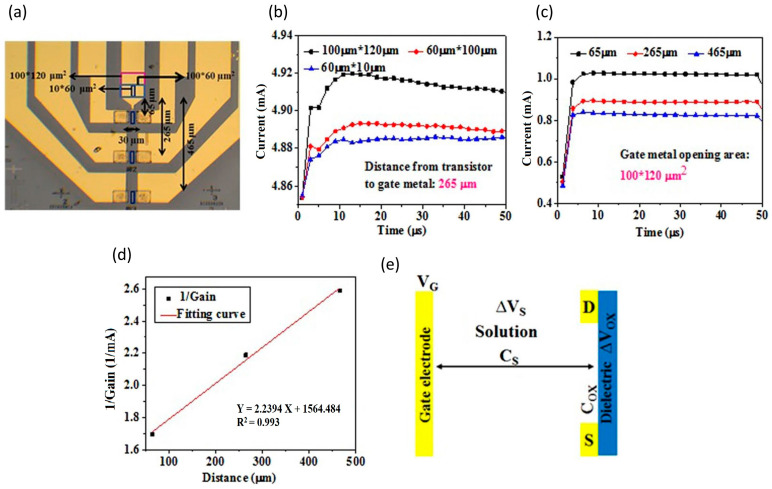
(**a**) EDL HEMT top view with several geometries created. Changes in drain current with comparison of (**b**) a different gate electrode opening regions and (**c**) and different gap geometries between gate opening and active channel. (**d**) Output gain current with increasing gap distance. (**e**) Schematic diagram of voltage drops across EDL HEMT. Reproduced with permission from reference [147]. Copyright 2017 Nature.

Chu et al. also developed a functionalized gate electrode of AlGaN/GaN HEMT EDL-based biosensors that exceeds the Debye length [147]. This sensing strategy can discriminate HIV1-RT from a variety of different concentrated solutions as low as 1 fM (detection limit). Remarkably, unlike typical FET biosensors, which are considered to detect proteins based on the net charge of proteins, their sensor is not reliant on the charges of proteins. One of the informative findings that the researchers could have concluded is that the sensor was investigated with a comparison of a different open area size on the gate electrode, as well as the distance gap between the gate electrode and the active channel, as shown in Figure 9a. The results in Figure 9b display the current gain increases towards larger open area size on the gate electrode. This is due to the fact that the bigger the gate electrode opening, the more ions are drawn to the gate electrode, resulting in additional ions collecting on the surface of the active channel, causing rising electron concentration in the channel and enhanced current output. Thus, the researcher concludes that this device is an ion-gated FET. Following the graph in Figure 9c, the results demonstrate an increasing distance gap between the gate electrode and the active channel, decreasing the current gain. This outcome indicates a correlation analysis with Figure 9d, as increasing gap distance is reversely proportional to current output gain. In other words, the sensitivity is enhanced when the spacing between the gate electrode and the channel is smaller since the sensing region creates less potential drop in the C_s_, which efficiently provides a larger drain current gain [62,148]. These results are consistent and repeatable, highlighting the reliability and capability of HEMT biosensors in physiological environments beyond the Debye length. Figure 9e shows schematic diagram of the voltage drop across an EDL HEMT. Notwithstanding the excellent performance of the AlGaN/GaN HEMT EDL-based biosensor, it would be interesting to see how this sensor performs with multiple detections in a single drop of blood.

**Figure 10:**
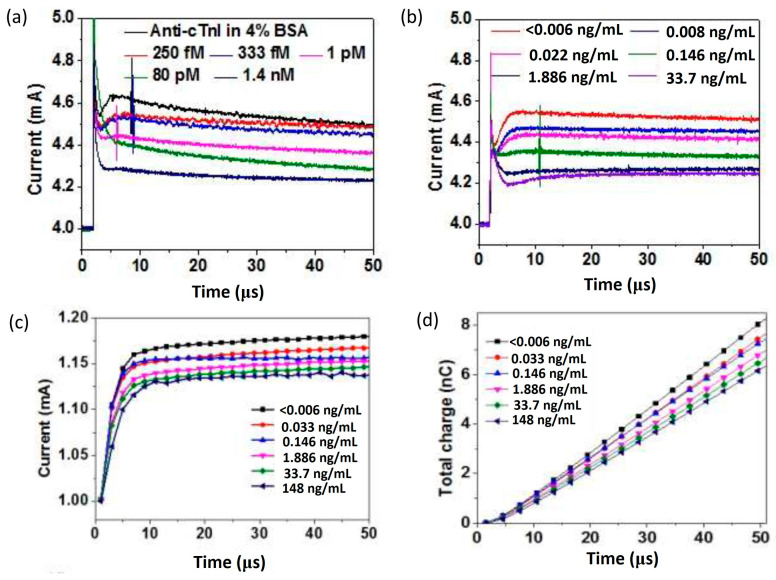
Current versus time graph of (**a**) purified proteins prepared in 1 X PBS containing 4% BSA and (**b**) clinical human serum samples for the antibody-based detection of Troponin I. (**c**) Current versus time and (**d**) total charge versus time graph for the aptamer-based detection of Troponin I in clinical human serum samples. Reprinted from [120], Copyright 2018, with permission from Elsevier.

Later, Sarangadharan et al. established an EDL FET-based biosensor for troponin I detection in 1 X PBS with 4% BSA and clinical human serum samples [120]. The use of both the anti-troponin I antibody and troponin I-specific aptamer as receptors has been analyzed in this work. The surface functionalization was performed with the native thiol groups in the monoclonal IgG molecule covalently binding to the Au gate, whereas thiolated aptamers formed SAM on the Au gate electrode. He stated that the aptamer was superior to antibodies because of the sensor’s stability, low cost, longer shelf life, and lesser deviation compared to differing sources of antibodies. However, the lesser immunoreactivity or affinity and a lack of availability of aptamers for all the disease biomarkers are some of the present drawbacks that make antibodies the primary choice for receptor. By comparing the antibody-based sensor results in Figure 10a,b, the outcome from testing in a purified buffer system and human serum samples exhibit similar drain current responses, and thus prove that the sensor can target specific antibody–antigen binding at the gate electrode interface. The sensor showed good selectivity towards troponin I despite the interferences from non-target proteins in the serum samples. Noteworthy, this sensor device’s operation did not require a washing procedure, which potentially could improve the sensor’s reliability. In order to enhance the sensitivity of aptamer-based sensor measurements, the total charge accumulated on the device surface is calculated by integrating the drain current through time, as illustrated in Figure 10c,d, so that random noise can be removed. It is apparent that, as the concentration of clinical human serum increases, the current also increases, thereby decreasing the total charge. These findings show that both antibody and aptamer-based sensors can achieve similar detection limits (<0.006 ng/mL). However, aptamer-based sensors exhibited a wider dynamic range from 0.006 to 148 ng/mL, which is more relevant to the current clinical concentration range of cTnI. Furthermore, this sensor accomplished troponin I detection in serum samples with a small sample volume (<2 µL) in just 5 min. This paper demonstrates its capability as an ideal candidate for enhancing the sensing technology for personal healthcare and disease management.

They further advanced the EDL gate HEMT biosensor by investigating clinical biomarkers for CVDs, including CRP, NT-proBNP, and cardiac Troponin I, using both an antibody and aptamer in a single chip [149]. By this means, both antibody and aptamer are covalently linked to the sensing region. The joint region of the thiolate ssDNA antibody is selectively cleaved using a mild reductant to fill half IgGs with native thiol groups to bind onto the gate electrode. The sensor demonstrates the LOD for each protein biomarker, which is 0.2 mg/L for CRP, 181 pg/mL for NT-proBNP, and 0.006 ng/mL for Troponin I, respectively. Besides, the total charge accumulation for CRP detection increases with increasing concentration, whereas the total charge decreases for NT-proBNP and Troponin I. The results are not affected by the total charge carried by these proteins because their isoelectric points do not correlate with the trend; instead, the different capacitive effects on the sensor were caused by protein–protein or aptamer–protein interactions. Regardless of this information, the effects of the related interactions are still unclear, including the dynamic range of detection and sensitivity of the sensor. Hence, further studies of receptor–ligand binding kinetics and optimal sensor bias conditions can be optimized for each biomarker.

## 6. Gateless HEMT

The basis of most HEMT biosensors is comprised of three terminal devices which are termed as the source, drain, and gate terminals. The detection of the HEMT biosensor is based on current changes as a result of surface potential changes caused by the analytes/biomolecules binding onto the sensing area. These analytes/biomolecules are drifted to the sensing surface by a voltage that is applied at the gate terminal (gate voltage). Nevertheless, the absorption of analytes/biomolecules onto the sensing area without the drive of the gate voltage is also possible [4]. The absorption of analytes/biomolecules onto the sensing area will change the sensor surface state and accordingly modulate the current flow in the two-dimensional HEMT channel in a manner analogous to that of a classic HEMT biosensor. Selective absorption of analytes/biomolecules on the sensing area can be obtained using various surface functionalizations, including conjugation with bio-recognition elements, self-assembled monolayers (SAMs), and ion-selective membranes (ISMs). Although eliminating the gate terminal (or the absence of a reference electrode) seems likely to simplify the HEMT sensor configuration, some of the studies reporting on biosensors based on gateless HEMT are certainly laborious, complex, and expensive procedures [43,150]. Aside from that, a major problem of electrode solution leakage continues to jeopardize the lifetime of devices and sensor performance [151].

Ma et al. modified the gate functionalization of AlGaAs/GaAs HEMT lactic acid sensor with indium (In)-doped zinc oxide (ZnO) nanowires [49]. The In atoms are doped in situ into the crystal structure of ZnO nanowires via the chemical vapor deposition (CVD) method. The diameters and lengths of the nanowires are in the range of 50 to 100 nm and roughly 10 μm, respectively. They measured the real-time lactic acid detection with changes of current within the source and drains under a constant bias voltage of 500 mV. The sensor exhibits good stability, and the current response is stable when kept in a PBS solution for approximately 200 s. Stability is a crucial criterion for the sensor, thereby excluding possible noise arising from the change in lactic acid solution, which is of key importance. The introduction of a target lactic acid, lactate oxidase (LOx), onto the surface sensor showed rapid detection of less than 1 s, reflecting the sensor’s specificity. Afterwards, the current stabilized after In-doped ZnO nanowires efficiently absorbed and immobilized with the LOx. This sensor successfully detects a wide detection range from 3 pM to 3 mM and has a low detection limit of 3 pM. This is because In-doped ZnO nanowires offer an effective surface area with a high surface area-to-volume ratio. The results of the sensing analysis were constant after five repetitions, suggesting that the sensor has good repeatability. Further, the author examined the performance of pure ZnO nanowires in a controlled experiment. The irregular and delayed response time of 10 s for every additional lactic acid concentration (from 3 pM to 3 mM) leads to inaccurate detection. This indicates that In-ZnO can enhance the conductivity of the HEMT sensor as well as abridge the electron transfer between the In-ZnO nanowires and the electrodes of the HEMT sensor. Furthermore, this In-doped nanowires afford a suitable environment for retaining the activity of LOx in comparison with pure ZnO nanowires. Since this HEMT sensor does not require a reference electrode, the amount of target is only dependent on the area of the sensing gate, which can improve selectivity in the sensor.

Taking advantage of a reference electrode-free structure, Myers and his group developed an AlGaN/GaN sensor for nitrate detection for the first time to examine sensing solely based on the ion activity [152]. In this work, they demonstrated polymer-functionalized AlGaN/GaN, containing a plasticizer and an ionophore to detect nitrate ions in solution. The device was equilibrated in 0.1 M KH_2_PO_4_, 0.1 M K_2_SO_4_ or 0.1 M KCl to test the device’s response toward nitrate ions. Their device demonstrated a rapid detection of less than 60 s after each addition of nitrate (30 min time intervals) as well as a stable response with a rms noise level of less than 0.3 μS. However, there is a slightly slower response after each addition, which occurs predominantly at higher concentrations, a phenomenon is due to solution mixing and membrane equilibration. Yet, this sensor still has good stability when the nitrate detection limit and linear range remained approximately the same between runs on separate days, even though there was variability in the response behaviour. The device achieves a detection limit of less than 1 × 10^−6^ M and a linear response range of 10^−6^–10^−3^ M in a 0.1 M KH_2_PO_4_ ion buffer, while 0.1 M K_2_SO_4_ and 0.1 M KCl ion buffers show 10^−6^ M and 10^−4^ M, respectively. The free electrode configuration prevents the specific stability/conditioning issues, allowing for greater miniaturization and lower manufacturing costs. This is not to mention that an array of sensors could easily be integrated into a single chip, enabling the detection of a large number of analytes. This work proves the possibility of extending this concept to a wide selection of ion-selective PVC-based membranes.

The detection of cardiac troponin I (cTnI) using AlGaAs/GaAs HEMT-based biosensors was proposed for the first time by Luo et al. in 2020 [9]. cTnI is a biomarker for the diagnosis of acute myocardial infarction (AMI). In this work, the sensor was functionalized by immobilizing the cTnI-antibody via SAM formation of 6-mercaptohexanoic acid to connect on the Au-thiol gate. The results of I_SD_-V_SD_ characteristics before functionalization show no significant effect on different concentrations of cTnI. However, after the gate functionalization, I_SD_ value decreased by a total of 11% as the concentration of the cTnI antigen increased. This was most likely because more cTnI biomolecules connect to the gate and influence the channel conductance. As a result, this highly sensitive biosensor effectively detects cTnI at comparatively low concentrations ranging from 1 pg/mL to 10 ng/mL and has a fast response time of less than 30 s. The proposed sensor exhibits current noise of less than 0.1 μA within 10 s before the current stabilizes approximately at 15–30 s. This result may be explained by the sudden drop of the cTnI, which causes intense changes in current due to the mechanical vibration of the solution. Regardless of the noise, this proposed sensor featured superior stability compared to the traditional measurement method (ELISA), which required approximately 1–2 h to stabilize the current. Further, the selectivity of this sensor was proven when the current drastically changed after cTnI antigens were released from the serum of AMI patients. It is consistent with the fact that the antibody binds specifically with the antigen. This discovery is remarkable because it simplifies sample purification procedures and reduces diagnostic time even further. The positive outcome realizes the capability of the GaAs HEMT biosensor to achieve high sensitivity and real-time detection of cTnI.

In order to improve sensing immobilization and target hybridization, Ding’s group evaluated a molecular-gated AlGaN/GaN HEMT pH sensor with SAMs of 3-aminopropyltriethoxysilane (APTES) as a transducer [153]. The sensing area is modified by APTES to provide amphoteric amine groups, -NH_2_, which are pH-sensitive, while the unmodified region may provide -OH, constituting the hybrid binding sites for pH detection. The results of the MG-HEMT sensor show good sensitivity and low current hysteresis with a resolution of 0.1 pH. This improvement mainly is caused by the predominant role played by amine groups, although the unimmobilized sensing area of GaN (without APTES, GaN exposed) still presents some hydroxyl groups for the detection. The shortcoming of the sensor is its repeatability. This is most likely because of undesired corrosion upon sensing application, which causes degradation of the stability and sensitivity of the device. The authors also demonstrate recovery techniques, including UV/O_3_ treatment, HCl soaking, and APTES modifications. They showed that the devices are able to recover, particularly for the APTES modification, and a pH sensitivity of 37.17 μA/pH was obtained. Detailed analysis and discussion of AlGaN/GaN HEMT channel engineering by photoelectrochemical (PEC) oxidation was presented by Xue et al. [154]. The PEC oxidation method is one of the more practical ways to introduce oxidation on the GaN cap layer in the channel area. With core-level spectroscopy analysis as evidence, the research group suggested that Ga_2_O_3_ was formed by surface oxidation after the PEC oxidation treatment. With successive increases in intensity of the percentage of oxygen on the channel area, resulting in a threshold voltage shift to the positive side from −3.46 V to −1.15 V. Their findings also show that the gate voltage (V_G_), corresponding to the maximum transconductance (g_mMAX_) position (V_G_|g_mMAX_), was also shifted from −2.6 V to −0.1 V, which is close to V_G_ = 0 V. The sensing response of the sensor device was carried out with a reference electrode and validated in pH solutions (pH 4, 7 and 10). There was a significant positive relationship between the pH and the current drain (I_D_). As the pH increased, the current drain of the sensor device also increased. An implication of these findings is the possibility that the proposed sensor could operate without the reference electrode, thereby making the miniaturization of the sensor possible.

## 7. Floating-Gate HEMT

**Figure 11:**
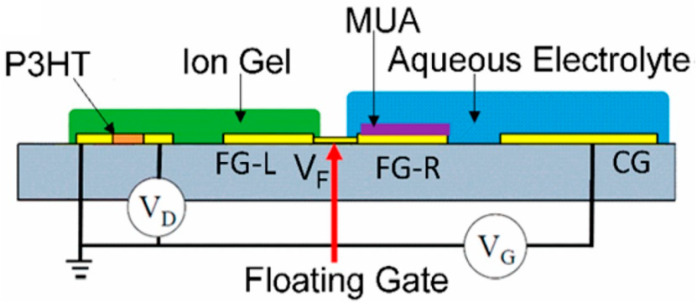
Representative diagram of the floating gate transistor. Reprinted with permission from [159]. Copyright 2018, American Chemical Society.

Principally, the floating-gate HEMT biosensor is fabricated with two-gate terminals, where one gate is used as the sensing gate and the other gate is used to apply control gate bias [155]. To aid proper understanding of the floating-gate HEMT biosensor, a cross-sectional floating-gate HEMT configuration is shown in Figure 11. In essence, a floating gate is electronically linked to the sensing active area of the transistor but physically detached from it. The primary advantage of floating-gate HEMT is the elimination of the reference electrode in the sensor system as the gate bias (V_G_) can be applied through the control gate terminal; therefore, the floating-gate HEMT can potentially be exploited as a miniaturized sensor [156]. The sensing mechanism of a floating-gate HEMT biosensor without a reference electrode can be elucidated as follows; the control gate has operational voltage applied that is high enough to turn ON the sensor device. The potential change caused by chemical/biological binding or biomolecule absorption onto the sensing gate consequently modulates the floating gate potential, leading to HEMT sensor voltage threshold (V_TH_) shift, suggesting the sensor’s response to the biological events [156,157,158]. This structure suggests that the performance on semiconductor/dielectric pair can be exploited rather than the stability of the sensing medium [159]. This structure can also prevent the sensing medium from contaminating the transistor channel [160,161,162].

**Figure 12:**
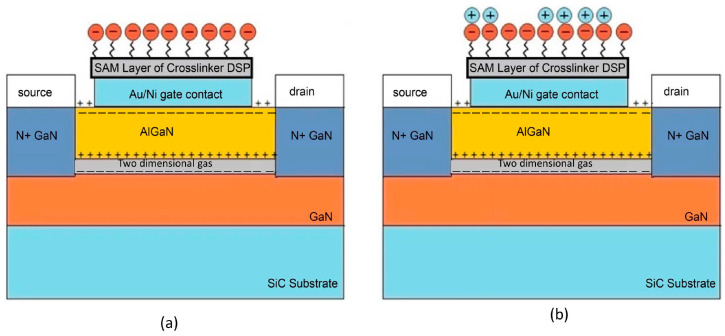
Schematic diagram of (**a**) immobilization of anti-MIG receptors and (**b**) conjugation of MIG antigen on the proposed sensor. Reprinted with permission from [165]. Copyright 2016, Journal of Modern Physics.

Tulip et al. and co-workers developed an AlGaN/GaN floating-gate HEMT biosensor for the detection of monokine induced by interferon gamma (MIG/CXCL9) [163]. MIG/CXCL9 is an immune biomarker for early monitoring of transplant or allograft rejection. In this work, the research group utilized short N-hydroxysuccinimide-esters, functionalized with bisymmetrical disulphide (DSP) to form reactive self-assembled monolayers (SAMs) for the immobilization of high affinity anti-MIG monoclonal receptors on the gold sensing gate of the HEMT device. The benefit of this sensing strategy is that it can eliminate chemical activation steps in the covalent attachment of biomolecules, as well as additional layers of chemical activating groups that are usually practiced in conventional methods. The short height of DSP SAMs (spacer arm length of 12.0 or 8 atoms) was favoured for HEMT biosensing as it provided close proximity of the binding antibody–antigen pair to the sensing gate surface, resulting in sensitive detection of the binding event. The same approach has been used recently by Butterworth et al. [164], who investigated the SAM composition in relation to DNA biosensor performance for antibiotic resistance. In his article, Butterworth concluded that the shorter SAM molecules increase the sensitivity of the sensor device. Despite its promising potential for application in high sensitivity sensors, short SAM molecules are less stable compared to long SAM molecules due to a lack of van der Waals attraction force [3]. Thereby, it is reasonable to think that the shorter SAM may show an ageing effect when the sensor is washed or regenerated. These effects may be disregarded for laboratory test purposes. However, this sensing strategy may not be appropriate for point-of-care testing and reusable sensors. On top of that, the authors also implemented the Schottky gate HEMT, which operates in the saturation region as opposed to the sub-threshold region operation of the insulated gate HEMT. Although the sub-threshold region operation can offer low-power application, the saturation region operation allows for a larger current change compared to sub-threshold region, which has the benefit of achieving higher sensor sensitivity. The floating gate configuration (which requires no voltage supply at the gate terminal) that was used in this work plays an important role in the sensor’s sensing accuracy. The change in drain current was solely dependent on the binding event of biomolecular MIG/anti-MIG with no gate voltage influence at the gate terminal. The sensor successfully detects MIG for a wide range of concentrations varying from 5 ng/mL to 500 ng/mL. This device’s reproducibility is demonstrated by the fact that the results of three test data sets at a 5 ng/mL MIG concentration fall within the minimal standard error. In a different study, Huq et al. [165] compared AlGaN/GaN HEMT sensing performances using numerical model simulation via SILVACO with measured experimental data. Similar to the study by Tulip et al., DSP SAM was developed onto the gold-plated floating gate to accommodate anti-MIG receptors to detect MIG target biomolecules. Figure 12a,b illustrate immobilization of anti-MIG receptors and the conjugation of MIG antigen onto the proposed sensor, respectively. Upon binding with the DSP, an increase in drain current is expected as the anti-MIG carries negatively charged ions, consequently altering the positive surface charge potential and resulting in a change in sheet carrier concentration in the hetero-interface. The V_TH_ of the simulated device and the actual device achieved are the same (V_TH_ = −4 V), however, there is an inconsistency in saturation drain current. The actual device demonstrated higher saturation drain current compared to the simulated device. This discrepancy could attribute to the fixed carrier concentration approximation (disregard trapping effects in 2-D simulation). While their simulation of the effects of protein creation and the immobilization of the SAM layer revealed an increase in current of 80 μA, the experimental results also showed an 80 μA increase in current upon the construction of the SAM layer on the floating gate and decrease in 70 μA upon the introduction of the target protein on the gate surface. The decrease in current was reasonable due to the interaction of anti-MIG with MIG target. The positively charged MIG target paired with the negatively charged anti-MIG, resulting in neutral charges at the gate surface, which thus altered the conductivity of the channel. To summarize, the simulation results were in good agreement with the actual experimental results, with minor variation around a few parameters. However, the authors failed to acknowledge the significance of current changes when the anti-MIG binds with the MIG target in the simulation.

Varghese et al. reported a mathematical model for the floating gate ofAlGaN/AlN/GaN HEMT for the detection of c-erbB-2 protein as a biomarker for breast cancer [166]. The primary goal of their work was to enhance the sensitivity and long-term stability of an HEMT device in order to make it feasible and precise for the detection of biomolecules in saliva and human serum. The model was simulated using SILVACO ATLAS TCAD. In favour of improving the HEMT sensor, the author has proposed the utilization of an ultra-thin A1N spacer layer with a thickness of 1 nm between the AlGaN and GaN layers to serve as a link between the device and its sensing area. The role of the AlN spacer in this HEMT biosensor was to improve the 2DEG density by excluding the charge carriers from the barrier AlGaN layer [167,168]. After the investigation into the effect of the channel/2DEG modulation relationship with the biomolecules, immobilization was done using floating-gate HEMT to prevent nullification by the applied gate bias. The researchers reported that the addition of an AlN interlayer to the epitaxial design has improved the device’s ON current and sensing currents beyond those of conventional HEMT sensor designs. Further, the authors investigated the effect of gate length on sensor performance. One unanticipated finding was that the device ON current and sensitivity of the HEMT sensor decreased as the gate length increased. In a practical situation, the device performance should increase with the gate length as the bio-immobilization area increases. However, in this case, it may seem like the result is contrary to what was expected. These may be due to the modeling/simulation limitations, whereby the method considers an approximate uniform sheet charge, while bio-concentration/distribution is more discrete in real cases. As the gate length increased from 1 to 5 μm, the device ON current decreased from 1.97 A/mm to 0.48 A/mm, and the sensitivity of the sensor for detection of c-erbB-2 concentration of 12 μg/mL also decreased from 2.504 to 0.72 mA/mgL^−1^. Nevertheless, this sensor’s sensitivity performance at 5 μm gate length was much higher than that of the other FET biosensors. Having said that, a device with a longer gate length is likely to have better stability as it results in higher breakdown voltages and lower noise levels. This study has provided new insights as a first-time mathematical model of spacer-based AlGaN/AlN/GaN HEMT, which is analyzed for biosensing applications. 

## 8. Dual-Gate HEMT

**Figure 13:**
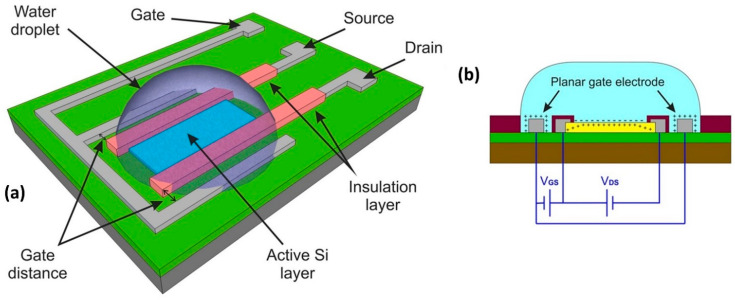
An illustration of (**a**) 3-D dual-gate setup and (**b**) schematic diagram of dual-gate setup. Reprinted with permission from [170]. Copyright 2017, Springer Nature.

For the amplification of the signal, dual-gate FET-based biosensors have gained attention as they only require a simple structural modification [169]. As the name implies, the structure has an additional gate in contrast with a conventional structure. A dual-gate structure consists of a bias gate (supporting bias) for the sensor to use in order to operate in a sensitive region, in addition to the use of the sweeping gate to measure the voltage signal. In contrast to conventional FET biosensors that are activated by a single gate, symmetric/asymmetric biases can be applied to two gates in the dual-gate FET biosensor. This enables an independent, precise, and tunable control of bias, which could remarkably improve the sensitivity of the biosensor. There are two types of dual-gate biosensors, one with the additional gate positioned at the bottom of the device, and the other with the additional gate positioned side by side on the same plane. The latter is called a planar dual-gate structure, as shown in Figure 13a,b. It should be noted that it is indeed more complex and challenging to fabricate back-gate structures for HEMT biosensors than it is for devices based on silicon and its oxides. 

Thus, up until now, there has been no report made on a dual-gate HEMT-based biosensor with the back gate. On the other hand, the planar dual-gate AlGaN/GaN HEMT structure has been developed by Cheng’s group for pH detection, which is comparable to a cascode amplifier [171]. The device consists of two gates on the AlGaN surface, which correspond to two single-gate HEMTs connected in series. The dual gates are constructed in circular shapes, of which the radiuses of the two gates are 750 μm and 850 μm, respectively, and they are connected in a cascode circuit. A constant bias was applied to the first gate (V_G1_), while the sensing gate bias (V_G2_) was regulated through the liquid via the quasi-reference electrode. The V_G1_ is passivated with SU-8 photoresist, and the V_G2_ is exposed to pH solutions for the detection of H^+^ concentrations. This offers the benefit of a pH sensor with adjustable sensitivity. This was proven by the fact that the increment in pH sensitivity was approximately 45 times higher when two different controlled constant output biases were applied. When the constant output bias was 1 V, the sensitivity was only 0.045 V/pH, however, at −4.36 V, the sensitivity dramatically increases to 2.06 V/pH. These remarkable findings verify that the dual-gate mode can achieve a tunable and higher amplification effect in order to increase the pH sensitivity of HEMT biosensors. Furthermore, the device’s pH sensitivity can be maximized further linearly with V_G1_, and its maximum voltage is limited by the resistance, R_D_ due to its tunable amplifier gains. Hence, for high sensitivity, when the range of pH detection is small, the sensor can be calibrated for high amplification with a large output resistance and a high metal gate voltage to identify the smallest variations. Similarly, it can be calibrated to have a significantly lower sensitivity for detecting a wide pH range with a small resistance and a lower metal gate voltage. The proposed design enables the improvement of HEMT biosensor sensitivity at the point of architecture instead of in subsequent complex amplifier circuits.

## 9. Challenges and Opportunities of Heterojunction-Based HEMT

Heterojunction-based HEMT has enormous benefits as a biosensor, but the pace of advancement has come with a baggage of obstacles in its actual manufacturing realization. One major issue is the influence of the Debye screening effect, a phenomenon that occurs in high ionic strength solutions and that results in extremely low Debye length, λ_D_. This high ionic strength solution includes urine, whole blood, and serum or physiological solutions. Detection of biosensors through changes in charge distribution and potential gradients is limited only within the λ_D_ [141]. Typical physiological samples have small dimensions (about 0.7–2.2 nm) in comparison to the size of larger receptor molecules like antibodies (about 10–15 nm). Thus, in high ionic strength solutions, the physiological samples that exceed the λ_D_, the shifts in charge distribution and potential gradients may not be verified. This severely limits antigen detection utilizing biosensors [172]. Hence, the most common method to rectify this issue is by significantly reducing the ionic strength of solutions by desalting or diluting the solutions to overcome the Debye screening effect [173]. This, however, requires further complicated sample pre-treatments [174] and may change biomolecule composition, resulting in the loss of target analyte activity and binding affinity [175]. Due to this issue, the true capability for detection of all FET-based biosensors, including HEMT, is limited since the direct detection of clinical samples is unfeasible [176]. Fortunately for HEMT biosensors, inspired by a new type of FET gated by ionic electrolytes, an electric double-layer (EDL) gate has been employed as a unique biosensing platform to suppress Debye length and enable direct detection in a physiological sample. Unlike traditional HEMT biosensors design, the biomolecule receptor is immobilized on a gate electrode that is separated from the active region. The issue of the Debye screening effect transformed into an advantage as the EDL formed the solution capacitance that controlled the current through the channel. A higher ionic strength solution augments the capacitance, which increases the current gain and sensitivity of the HEMT biosensor. Chen et al. proposed an EDL-gated HEMT sensor with probe DNA to capture target DNA from a physiological salt environment. The detection limit of the sensor can be as low as 1 fM with very high sensitivity [141]. 

The fabrication cost of heterojunction-based HEMT biosensors is the elephant in the room, hindering the sensor market’s breakthrough. In contrast to Si technology, heterojunction-based HEMT devices are still in their infancy [177]. Inexpensive disposable biosensor chips are still in demand on the market, whereas the currently available sensors on the market are still unfavourable due to their limitations and problems with false positives. There is still a need for a highly sensitive and reliable sensor, which HEMT biosensors simply offer. The process of device fabrication of these HEMT biosensors also remains complex, time-consuming, and expensive [178]. For instance, the deposition of a gold layer as a surface functionalization increases the processing cost, however, the use of nanostructured gold materials such as gold nanoparticles [179] or gold nanoislands [180] minimizes the cost. Captivatingly, the integration of nanomaterials and HEMT further enhances the sensor properties. These are not limited to response and sensitivity, as the method enables multiplex detection for preclinical and clinical applications [181]. Multiplex detection is highly desirable, as only a single specimen sample and biosensor are required to detect multiple desired targets simultaneously rather than having to use multiple samples and biosensors for each target separately. Moreover, multiplex detection would also delineate and distinguish between closely related targets that are present and have similar properties or symptoms. These would provide cost-effective fabrication solutions for HEMT biosensors.

## 10. Conclusions

This review paper introduces the sensing mechanisms and various gate structures of the heterojunction-based HEMT biosensor in developing better performance and overcoming the sensor’s limitations. The sensitivity, selectivity, specificity, reliability, repeatability, stability and reproducibility of the biosensor have become crucial parameters in order to determine an excellent biosensor. Through surface modification to the sensing surface, the conventional electrolyte-gate HEMT exhibits great sensitivity. It does, however, have limited detection in certain conditions. The extended-gate HEMT structure offers reliable biosensors at a low cost of manufacturing, with the ability to be reused since the transducer and sensing area are entirely isolated. The EDL gate structure overcomes the Debye screening effect, allowing for the detection of biomolecules in high ionic strength solutions with high sensitivity. HEMT biosensors benefit from high selectivity, even with low operational voltage since the device mechanisms are solely based on 2DEG mobility channels rather than the high ionic strength of solutions with an electrolyte gate structure. The use of a dual-gate HEMT structure as a biosensor enables the amplification of sensitivity and controlled selectivity. With considerably cost-effective HEMT biosensors, multiplex detection ability, and integration of advanced microfluidic systems, this brings us to the current interest in the implementation of HEMT biosensors in the ecosystem of the Internet of Things (IoT). Accordingly, with the Fourth Industrial Revolution merging the biological with the digital world, it is of the essence to move forward.

## Figures and Tables

**Table 1:** The characteristics of several biosensors for detection of CRP, BNP, PSA, HER2/C-erB-2 and CA 19-9.

Measurement Technique/ Device	Detection Layer	Medium	Range	Limit of Detection	Response Time	References
***C-Reactive Protein (CRP)***						
HEMT	CRP-specific aptamer	Human serum	0.24–1.18 mg/L	0.34 mg/L	10 µs	[10]
HEMT	CRP-specific aptamer	Clinical sample (serum)	0.029 mg/L–2900 mg/L	0.029 mg/L	10 min	[55]
HEMT	CRP-specific aptamer	Clinical sample (serum)	0.625 mg/L–10.000 mg/L	0.34 mg/L	50 µs	[13]
FET	Cysteine-tagged protein G + Anti-CRP	PBS	3–20 mg/mL	0.01 µg/mL	10 min	[56]
Electrochemical	AuNPs + Anti-CRP	Human serum	0.4–200 nM	0.15 nM	30 min	[57]
Electrochemical	Copper NPs	Human serum	1.0 fg/mL–100 ng/mL	0.33 fg/mL	45 min	[58]
Electrochemical	Bismuth citrate	Human serum	0.2–100 ng/mL	0.05 ng/mL	30 min	[59]
Fluorescence	Monoclonal antibody + QDs	Human serum	0.5–300 mg/L	0.25 mg/L	15 min	[60]
SPR	Anti-CRP	Human serum	1 ng/mL–10 µg/mL	10 pM	>1 h	[61]
***Brain Natriuretic Peptide (BNP)***						
HEMT	Aptamer	Clinical serum	0–10 ng/mL	-	5 min	[62]
HEMT	Anti-BNP on microbeads	PBS	0.47 ng/mL–1.29 pg/mL	97 fg/mL	5 min	[63]
FET	In_2_O_3_ nanoribbon + Anti-BNP	PBS	10–90 pg/mL	10 pg/mL	45 min	[64]
Electrochemical	AuPd nanocrystals/N-doped porous carbon (AuPd NCS/NPC)	PBS	0.001–10 ng/mL	0.34 pg/mL	-	[65]
Fluorescence	Anti-NT-proBNP	Human serum	200 pg/m/L–26,000 pg/mL	47 pg/mL	10 min	[66]
Fluorescence	GO + FAM-aptamer	Blood sample	0.074–0.56 pg/mL	45 fg/mL	-	[67]
SPR	Au nanocubes + Anti-BNP	Human serum	1 aM to 500 nM	1 nM	-	[68]
***Prostate Specific Antigen (PSA)***						
HEMT	Anti-PSA	PBS	0.1 pg/mL–1 ng/mL	0.1 pg/mL	150 s	[69]
FET	B-SA system with DNA tetrahedron	PBS Human serum	1 fg/mL–100 ng/mL	1 fg/mL	>2 min 4 min	[70]
Electrochemical	BPene + Au NPs	PBS	0.0001 ng/mL 10 ng/mL	30 fg/mL	-	[71]
Fluorescence	Sub-FAM	Human serum	1–100 pg/mL	0.76 pg/mL	60 min	[72]
SPR	Anti-PSA	PBS	0.5 pg/mL–500 pg/mL	1 pg/mL	5 min	[73]
***Human Epidermal Growth Factor Receptor 2 (HER2/C-erB-2)***						
HEMT	Au- HSCH_2_COOH + Anti-C-erB-2	PBS	0.25–16.7 µg/mL	0.25 µg/mL	>5 s	[74]
FET	Graphene nanomesh (GNM) + HER2 aptamer	PBS	0.0001 to 10 ng/mL	0.1 pg/mL	>10 s	[75]
Electrochemical	MWCNT(COOH)/AuNPs	Spiked human serum	7.5–50 ng/mL	0.16 ng/mL	-	[76]
Electrochemical	MIP/AuSPE	Spiked human serum	10 to 70 ng/mL	1.6 ng/mL	7 min	[77]
Electrochemical	(NFG)/AgNPs/PANI + Anti-HER2	Human serum	10^−5^ × 10^6^ cells/mL	2 cells/mL	30 min	[78]
Fluorescence	AgNCs (dsDNA-AgNCs) + HApt	PBS	8.5 fM to 225 fM	0.0904 fM	20 min	[79]
SPR	Anti-HER2 + ssDNA aptamers	PBS	10^−12^ g/mL–10^−6^ g/mL	9.3 ng/mL	-	[80]
***Carbohydrate antigen 19-9 (CA 19-9)***						
HEMT	APTES + Anti-CA 19-9	PBS	15 U/mL–150 U/mL	15 U/mL	-	[81]
Fluorescence	Carbon quantum dots/gold (CQDs/Au) + Anti-CA 19-9	Human serum	0.01–350 U/mL	0.007 U/mL	15 min	[82]
FET	MoS_2_ nanosheets + Anti-CA 19-9	PBS	1 × 10^−12^ U/mL–1 × 10^−4^ U/mL	2.8 × 10^− 13^ U/mL	20 min	[83]
Electrochemical	Au NPs + Anti-CA 19-9	PBS	0.1–10.0 µU/mL	0.030 µU/mL	-	[84]

**Table 2:** Reproducibility rate of HEMT-based biosensor. Relative standard deviation (RSD).

HEMT Platform	Detection	RSD (%)	Ref.
AlGaAs/GaAs	Oligoasthenospermia	<0.7	[85]
AlGaN/GaN	pH	0.5	[86]
AlGaS/GaAs	DNA	6.02	[87]
AlGaN/GaN	Phosphate anion	4.4	[88]
AlGaN/GaN	CRP	9.2	[89]

**Table 3:** Recent HEMT-based sensor for biosensing application.

Type of HEMT	Sensor Applications	Functionalization, Techniques	Sensitivity, Limit of Detection	References
AlGaAs/InGaAs	Mercury (II) irons (Hg^2+^)	Au-thiol ssDNA	10 nM	[105]
AlGaN/GaN	pH	Al_2_O_3,_ Ultrasonic spray pyrolysis deposition (USPD)	55.6 mV/pH	[106]
AlGaN/GaN	pH	Thermal oxidation treatment	57.7 mV/pH	[107]
AlGaN/GaN	pH	Al_2_O_3,_ Atomic layer deposition (ALD)	57.8 mV/pH	[108]
AlGaN/GaN	pH	Al_2_O_3,_ Atomic layer deposition (ALD)	-	[109]
AlGaN/GaN	Circulating tumor cells (CTCs)	Au-thiol aptamer	-	[110]
AlGaN/GaN	pH	Au	55 mV/pH	[111]
AlGaN/GaN	pH	Photoelectrochemical (PEC)	56.3 mV/pH	[112]
AlGaN/GaN	pH	-	54.38 mV/pH	[113]
AlGaN/GaN	pH	Ammonium hydroxide (NH_4_OH) treatment	84.39 µA/pH	[114]
AlGaN/GaN	Glucose	APTES SAMs	3.15 × 10^4^ µA/mM^1^cm^2^, 10 nM	[115]
AlGaN/GaN	Glucose	APTES/AuNPs	1 × 10^6^ µA/mM^1^cm^2^, 1 nM	[54]
AlGaN/GaN	Urea	APTES/AuNPs	18.15 mA/pC_urea_, 25 μM–50 mM	[116]
AlGaN/GaN	pH	-	69.5 mV/pH	[117]
AlGaN/GaN	pH	-	162 mV/pH	[118]
AlGaN/GaN	pH	-	132 mA/mm-pH, 950 mV/pH	[119]

## Data Availability

Not applicable.
